# Effects of floorball and strength training in a real-life setting on health and physical function in older men

**DOI:** 10.3389/fragi.2025.1574084

**Published:** 2025-07-25

**Authors:** Mogens T. Pedersen, Jens Bangsbo

**Affiliations:** Department of Nutrition, Exercise and Sports, Centre of Team Sport and Health, University of Copenhagen, Copenhagen, Denmark

**Keywords:** aging, bone health, exercise, physical capacity, team sport

## Abstract

**Background:**

There is lacking information about the physiological response when conducting exercise training of older men in a real-life setting.

**Objectives:**

The aim of this study was to examine the effects of floorball and strength training in a real-life setting on health and physical function in older men.

**Methods:**

Seventy-six sedentary men aged 72.3 ± 0.6 (means ± SE; range: 63–92) years with a height, body mass and body mass index of 178.9 ± 0.8 cm; 92.1 ± 2.2 kg: 28.7 ± 0.6 kg/m^2^, respectively, were recruited to floorball (FG, n = 29), strength (SG, n = 38) or bowls (BG, n = 9) training 1 h twice a week in municipal activity centers and senior sport clubs. Subjects were tested at baseline, after 12 and 24 weeks.

**Results:**

Twelve weeks of floorball and strength training lead to reduced (P < 0.05) blood glycosylated hemoglobin (Hb1Ac), body mass, fat mass, visceral and android fat. Further, SG had a decrease (P < 0.05) in gynoid fat as well as total and LDL plasma cholesterol. FG and SG decreased heart rate at rest. In SG, systolic and diastolic blood pressure were also reduced (P < 0.05). FG increased (P < 0.05) markers for bone growth. FG and SG improved (P < 0.05) functional capacity. The improvements in FG and SG were maintained after 24 weeks. BG did not have any changes.

**Conclusion:**

In conclusion, older men conducting floorball or strength training twice a week in a real-life setting can improve functional capacity and a high number of health factors, whereas playing bowls does not lead to physiological changes.

## 1 Introduction

Aging is associated with loss of muscle mass and decline in physical function. Thus, the ability to perform activities of daily living, such as climbing stairs and get up from a chair is reduced in elderly (Dutta, 1997; Foldvari et al., 2000; Cuoco et al., 2004). In addition, balance deteriorate with age, and the risk of falls and fractures increases (Faulkner et al., 2007). Aging entails also a number of health-related challenges, such as increase in body fat and blood lipids as well as decline in cardiovascular function with elevated blood pressure and reduced insulin resistance with a risk of development of type II diabetes (Houterman et al., 2002; Abdelhafiz and Sinclair, 2015).

Regular physical activity has been shown to improve functional capacity and the health profile of elderly (Greig et al., 1993; Rantanen et al., 1997; Bottaro et al., 2007; Lovell et al., 2011; Gliemann et al., 2013; Schmidt et al., 2014; Silva et al., 2014; Andersen et al., 2016; Chen et al., 2017; Sousa et al., 2017; Sun et al., 2018; Sbardelotto et al., 2019). Thus, aerobic training of older adults has have favorable health effects such as an increase in high density lipoprotein (HDL) cholesterol and a decrease in plasma triglyceride, while a decrease in low density lipoprotein (LDL) cholesterol seems to be related to an accompanying weight loss (Durstine et al., 2002). Reduction in fat mass and increase in insulin sensitivity has also been seen in elderly with aerobic training (Borghouts and Keizer, 2000). Strength training in elderly has lowered total and LDL cholesterol as well as increased HDL cholesterol (James et al., 2016; Chen et al., 2017). In addition, strength training increase strength and improve physical function and cardiorespiratory variables as well as increases insulin sensitivity combined with decreases in glycosylated hemoglobin in older adults (Bottaro et al., 2007; Schmidt et al., 2014; Silva et al., 2014; Chen et al., 2017). However, changes in insulin sensitivity with strength training seem to be mostly related to eccentric training (Chen et al., 2017).

One of the challenges is to get older adults motivated to do aerobic or strength training and for them to maintain the training (Nielsen et al., 2014). In contrast, team sports have been shown to motivate and sustain the participants’ interest in continuing the training for years (Nielsen et al., 2014; Pedersen et al., 2017). Team sports, such as soccer and floorball conducted as small-sided games, have been shown to have similar effects on health of older adults as both aerobic and strength training. Body mass is reduced after a period with floorball (Vorup et al., 2017b) and soccer (Andersen et al., 2014) training, fat percentage, android and visceral fat are lowered with floorball (Pedersen et al., 2022), glucose tolerance is elevated with soccer training (Mohr et al., 2023), and lean body mass is increased with various team sport activities (Andersen et al., 2014; Vorup et al., 2017a). Furthermore cardiovascular health becomes better with regular floorball (Vorup et al., 2017b) and football (Schmidt et al., 2014) training, and also bone health and functional capacity are improved (Vorup et al., 2017b; Pedersen et al., 2022). However, most studies of the effect of training of older adults are performed in a controlled environment, and there is lacking information about the physiological response when conducting training in a real-life setting.

Many older adults are not sufficiently active. In particular, older men take to a lesser extent part in physical activities compared to women, which may be caused by existing offers not being attractive for older men. Floorball training can offer a motivating and social stimulating team activity for older men (Vorup et al., 2017b). In Denmark Floorball is performed in various sports clubs for the elderly but is not particularly widespread. Strength training and Bowls, though, are widespread in sports clubs and activity centers for the elderly. Bowls is a game similar to pétanque. The participants seem to be motivated by the development of skills during participation in the game. The level of physical activity during a game, however, seems to be low. The health effects of playing Bowls have not been studied.

Thus, the aim of this study was to examine the physiological adaptations in older men recruited to regular participation in floorball (whole body high load exercises) or strength (isolated high load exercises) training and compare to the effects of performing bowls (isolated low load exercises) training. It was hypothesized, that floorball training was superior to strength and bowls training when it comes to cardiovascular health, reduced body fat and glycosylated hemoglobin and increase in bone health (Durstine et al., 2002). The effect on physical function was expected to be equal in the floorball and strength training group, but superior to the bowls training group.

## 2 Materials and methods

### 2.1 Subjects

Seventy-six sedentary men aged 72.3 ± 0.6 (means ± SE; range: 63–92) years with a height, body mass and body mass index of 178.9 ± 0.8 cm; 92.1 ± 2.2 kg: 28.7 ± 0.6 kg/m^2^, respectively, took part in the study. The participants were recruited from municipal activity centers, senior sport clubs as well as through announcements in local newspapers. Most subjects were recreationally active (walking or cycling for transportation on a daily basis), but none had been involved in any type of regular exercise training for the past 10 years.

The study was approved by the Committee on Health Research Ethics, Region of Copenhagen (H-18047204), and conducted in accordance with the guidelines of the declaration of Helsinki. The subjects were informed of any risks and discomforts associated with the experiments before giving their written informed consent to participate in the study.

### 2.2 Study design

**FIGURE 1:**
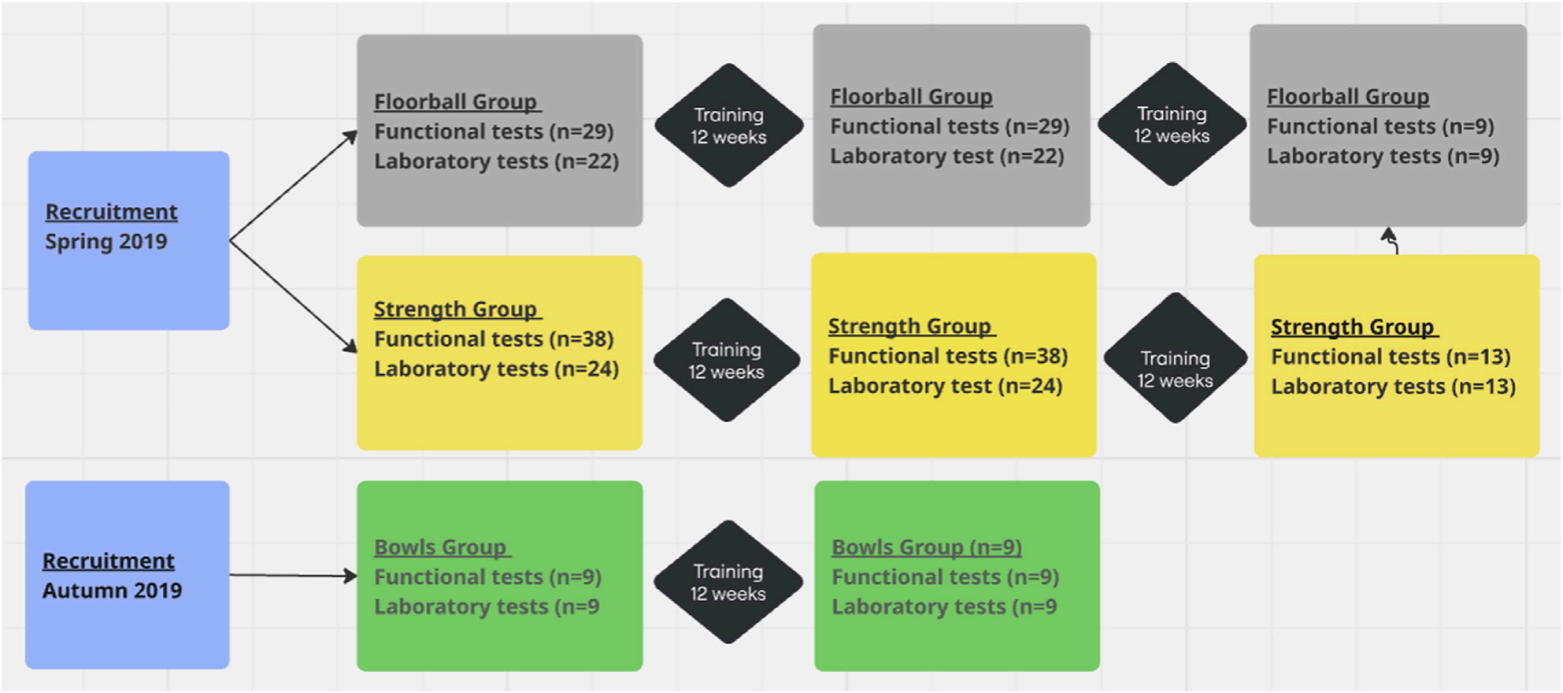
Flowchart.

In the spring 2019, the participants were recruited to participate in exercise training through announcements in local newspapers. In addition, some participants were recruited through e-mails to wives, who were active in sports clubs. Agreements were made with local sports clubs and municipalities to establish natural settings to conduct floorball, strength and bowls training. The participants could freely choose whether they wanted to participate in floorball, strength or bowls (comparable to indoor pétanque) training. Twenty-nine subjects chose floorball (FG), 38 subjects chose strength training (SG), whereas no one chose to play bowls (Figure 1). The participants were training 1 h twice a week during a 12-week intervention period (INT). After the 12 weeks, participants in FG and SG had the opportunity to continue their activity for another 12 weeks, and 9 and 13 subjects, respectively, continued for another 12 weeks (Figure 1). In the fall 2019, we succeeded in recruiting nine subjects to play bowls (BG) for 12 weeks in a sport club (Figure 1). The subjects conducted a number of tests (see below) before, and after 12 and 24 (except for BG) weeks of training. In addition, on a separate day before, and after 12 and 24 (except for BG) weeks of training 19 participants from FG, 22 from SG and 9 from BG, completed a number of physiological measurements (see below). During the study period, the participants maintained their habitual life routines and level of physical activity.

Some of the participants took medicine with the most frequent type being diabetes medication (12%), blood pressure medication (47%), cholesterol lowering medication (22%), anticoagulants (18%), and heart medication (15%). In BG, more subjects were taking medication for heart function, diabetes and cholesterol-lowering, compared to FG and SG (33, 29, 67% in BG compared to 9, 14, 8% in FG and 14, 12, 14% in SG respectively). There were no significant changes in medication during the 24-week intervention period.

There were no differences between groups at baseline for age, height, body mass index (BMI), blood pressure (BP), android fat, gynoid fat, visceral fat, blood HBA1c and high density lipoprotein (HDL) cholesterol, but differences between groups at baseline for four physical tests as well as plasma low density lipoprotein (LDL) cholesterol and glucose were observed (Table 1).

**TABLE 1:** Baseline data for the floorball training group (n = 22), the strength training group (n = 24) and the bowls training group (n = 9). For the functional tests the number of subjects were 29 and 38 in the floorball and strength training group, respectively.

	Floorball	Strength	Bowls
Age (years)	71.1 ± 0.8	73.0 ± 0.8	72.8 ± 1.6
Height (cm)	179.8 ± 1.3	179.0 ± 1.3	176.2 ± 1.9
BMI (kg/m^2^)	28.7 ± 0.9	27.8 ± 0.8	31.2 ± 2.3
Android fat (%)	44.2 ± 2.0	43.9 ± 1.6	47.9 ± 3.1
Gynoid fat (%)	31.3 ± 1.2	31.0 ± 1.3	37.4 ± 3.2
Visceral fat (kg)	2.47 ± 0.25	2.35 ± 0.22	2.63 ± 0.39
Diastolic BP (mmHg)	83.4 ± 2.3	88.2 ± 2.2	88.7 ± 2.7
Systolic BP (mmHg)	133.9 ± 3.9	146.6 ± 4.9	138.4 ± 3.8
LDL cholesterol (mmol/L)	3.4 ± 0.2	3.9 ± 0.2^β^	2.6 ± 1.8^γ^
HDL cholesterol (mmol/L)	1.4 ± 0.1	1.4 ± 0.1	1.4 ± 0.1
HBA1c (mmol/mol)	37.0 ± 1.1	38.0 ± 0.9	39.7 ± 2.7
Glucose (mmol/L)	6.1 ± 0.2^β^	6.0 ± 0.2^β^	7.5 ± 0.6^α,γ^
6-min walk (m)	567 ± 12^β,γ^	521 ± 13^α,β^	447 ± 24^α,γ^
Rise & Sit 30 s (reps)	12.0 ± 0.3^β^	11.5 ± 0.3	9.2 ± 1.9^α^
2.45 Up&Go	6.0 ± 0.2^β^	6.6 ± 0.3	7.6 ± 0.8^α^
Arm flexion (reps)	19.2 ± 0.6^β^	18.3 ± 0.6^β^	13.1 ± 0.9^α,γ^
Handgrip (KP)	43.2 ± 1.2	40.7 ± 1.4	36.1 ± 2.1

Values are mean ± SE., Hb1Ac, glycosylated hemoglobin; HDL, high density lipoprotein; LDL, low density lipoprotein; BP, Blood pressure. ^α^Significant (p < 0.05) different from the floorball training group; ^γ^Significant (p < 0.05) different from the strength training group. ^β^Significant (p < 0.05) different from the bowls training group.

### 2.3 Training

For each activity, a thorough instruction of how to conduct the activity, including rules for floorball and bowls, was made before starting the training.

#### 2.3.1 Floorball training

Floorball is a team sport like field hockey but played indoors with plastic sticks (http://www.floorball.org). All sessions started with a 10-min warm-up period, including mobility, stretching, and technical exercises. The participants completed a small-sided game with three to five players in each team. The court size was adapted to the physical function of the participants (the better the function, the larger the court). In the first part of the intervention period, the participants completed 4-min playing intervals separated by 4 min of rest. During the first 4 weeks (weeks 1–4), four 4-min intervals were conducted, i.e., total training time was 16 min per session. In the following 4 weeks (weeks 5–8), the participants completed six 4-min intervals, i.e., total training time was 24 min per session, and in the last 4 weeks (weeks 9–12), eight 4-min intervals were performed with a total training time of 32 min.

#### 2.3.2 Strength training

At each session, participants in the strength-training group conducted a 10-min warm-up session, including mobility, stretching and low load strength exercises with a technical focus. The strength-training program consisted of eight exercises focusing on leg, arm and core muscles. In the first 4 weeks, the participants performed 2 times 10 repetitions of each exercise with a low load separated by 2 min of rest. In the last 8 weeks, the participants completed 3 times 10 repetitions with an increasing load according to the individual’s progress.

#### 2.3.3 Bowls training

Bowls is a ball game that is similar to pétanque. However, unlike pétanque, bowls is primarily played indoors. Bowls is a technical and strategic precision game that involves placing the team’s own balls as close to the target ball as possible, and at the same time shooting away the opponent’s balls. In bowls, the balls are asymmetrical in weight. Bowls were typically played 3 vs. 3 or 4 vs. 4. A bowls-training session lasted approximately 90 min.

#### 2.3.4 Training compliance

In FG, SG and BG training compliance was 76.6% ± 3.5%; 76.5% ± 3.8% and 87.0 %± 8.2%, respectively, corresponding to ∼1.6 training sessions a week, with no difference between groups. Training compliance was not registered in the second 12-week intervention period.

### 2.4 Measuring and test procedures

Subjects were instructed to refrain from strenuous exercise for at least 36 h before testing and physiological measurements. Subjects on medicine were instructed to take their habitual medicine on experimental days.

#### 2.4.1 Functional capacity tests

At the training facilities, six standardized functional exercises were performed, including (a) maximal number of sit-to-stand repetitions in 30 s, (b) time to stand, walk 2 × 2.45 m (out and back around a cone) and sit (2.45 Up and Go), (c) maximal distance in a 6-min walking test, (d) maximal number of repetitions of biceps-curls with an 8 kg dumbbell (Rikli and Jones, 2013), (e) maximal hand-grip strength with an adjustable hydraulic hand dynamometer (JAMAR; North Coast Medical, Oakleigh, Victoria, Australia). The tests were performed indoors on a wooden surface.

#### 2.4.2 Additional experimental day

Subjects reported to the laboratory between 07:00 and 10:00 a.m. after an overnight fast. A blood sample was taken from a cubital vein for determination of plasma HDL, LDL and total cholesterol, glucose, HbA1c and bone markers [osteocalcin, procollagen type-1 amino-terminal propeptide (P1NP) and carboxy-terminal type-1 collagen crosslinks (CTX)]. P1NP and osteocalcin are markers for bone growth, and CTX is a marker for bone degradation (Helge et al., 2014).

Body composition was determined by whole-body dual-energy X-ray absorptiometry (DXA) scanning (Lunar Prodigy Advance; GE-medical Systems, Madison, Wisconsin, United States) and analyses by software (enCORE v15, GE-medical Systems, Madison, Wisconsin, United States). Then subjects rested at least 15 min in a supine position before blood pressure was measured six consecutive times by an automatic upper arm blood pressure monitor (M7; OMRON, Vernon Hills, Illinois, United States), while at the same time heart rate was measured (Polar Team System; Polar Electro Oy, Finland).

#### 2.4.3 Blood analysis

Blood samples were analyzed for total, HDL and LDL cholesterol, glucose, and HbA1c using turbidimetric immunoassay (Tosoh G8, Tosoh Bioscience Inc., South San Francisco, CA, United States) at the clinical biochemical unit at the Copenhagen main hospital (Rigshospitalet) using an automatic analyzer with enzymatic kits (COBAS 8000, Roche Diagnotics International Ltd., Rotkreuz, Switzerland). Plasma bone markers were measured by a fully automated immunoassay system (iSYS, Immunodiagnostic Systems Ltd., Boldon, England) by method of Chemiluminescence.

#### 2.4.4 Heart rate during training

At selected training sessions, subjects were wearing a heart rate monitor (Polar Team System; Polar Electro Oy, Finland) to measure heart rate. Heart rate data were subsequently analyzed using appropriate software (Polar ProTrainer 5, Polar Electro Oy, Finland).

### 2.5 Statistics

Comparisons of baseline outcome measures between FG, CG and BG were performed using ANOVA with *post hoc* Bonferoni Multiple Comparisons. The effect of floorball, strength and bowls training was evaluated by General Linier Model analyses including the final outcome measure as dependent variable and training type as fixed factor while adjusting for baseline values of the outcome. Crosstabs with Chi-square tests we used to look for differences in the distribution of medicine intake between groups. Analyses of changes were based on comparisons of date before and after 12- and 24-week training intervention period, as well as comparisons between data after 12 and 24 weeks. Distribution of the data was checked for normality before applying Annova and Linear regression. IBM SPSS statistics 25.0 was used for all tests. P < 0.05 was chosen as the level of significance and all data are presented as means ± SE.

## 3 Results

### 3.1 Effects of 12 weeks of training

#### 3.1.1 Heart rate during training

**FIGURE 2:**
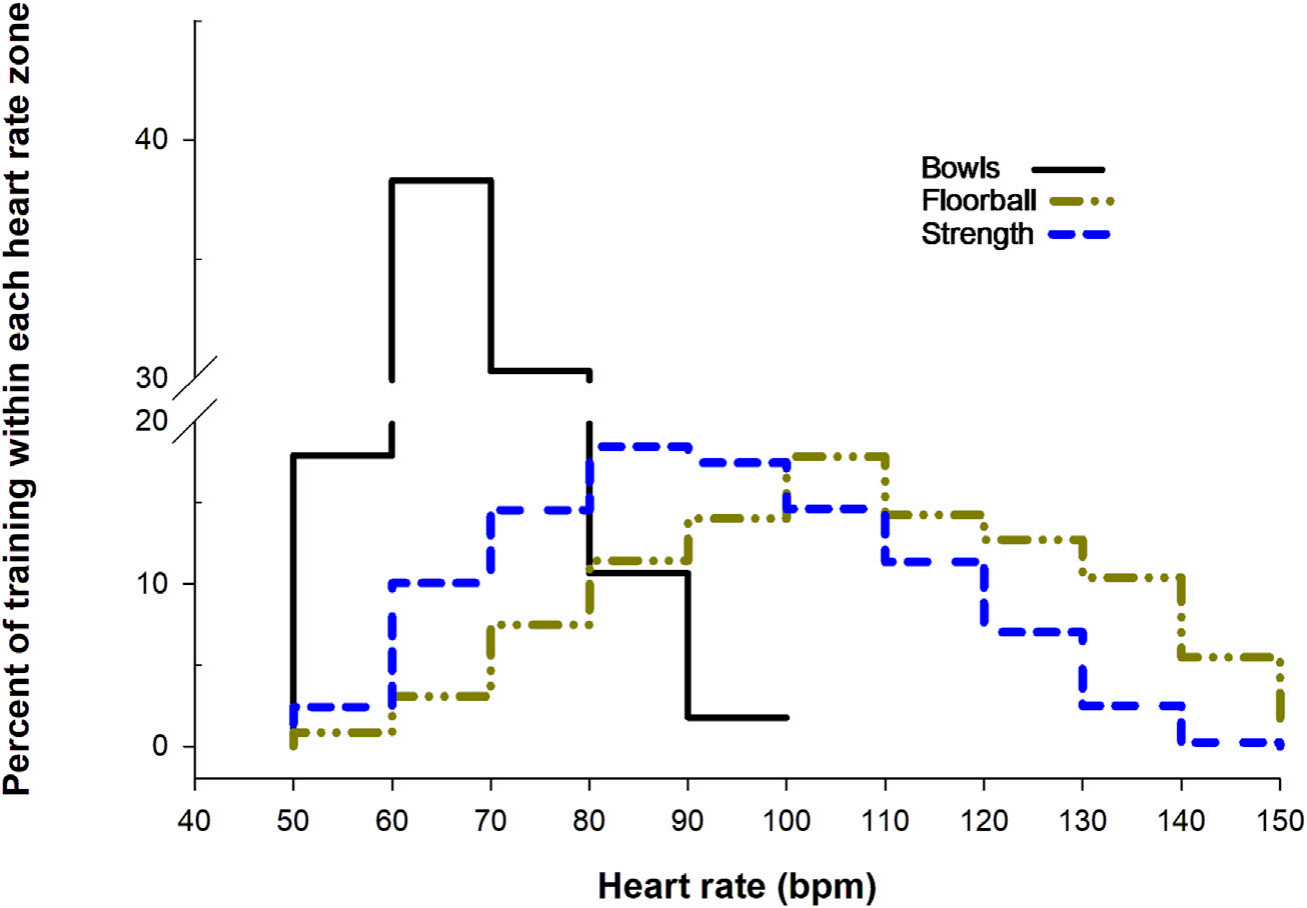
Heart rate distribution for one session of floorball training (68 min, n = 22), strength training (55 min, n = 24) and bowls training (88 min, n = 5). Data are presented as means within each heart rate zone (40–49, 50–59, 60–69 bpm, *etc.*).

Figure 2 shows the distribution of heart rate during one session of floorball, strength and bowls training. Mean and peak heart rate was 118 ± 4.6 and 149 ± 5.2 bpm, respectively, for floorball, 102 ± 3.2 and 133 ± 3.7 bpm for strength training and 84 ± 7.3 and 104 ± 8.8 bpm for bowls training.

#### 3.1.2 Body composition

**FIGURE 3:**
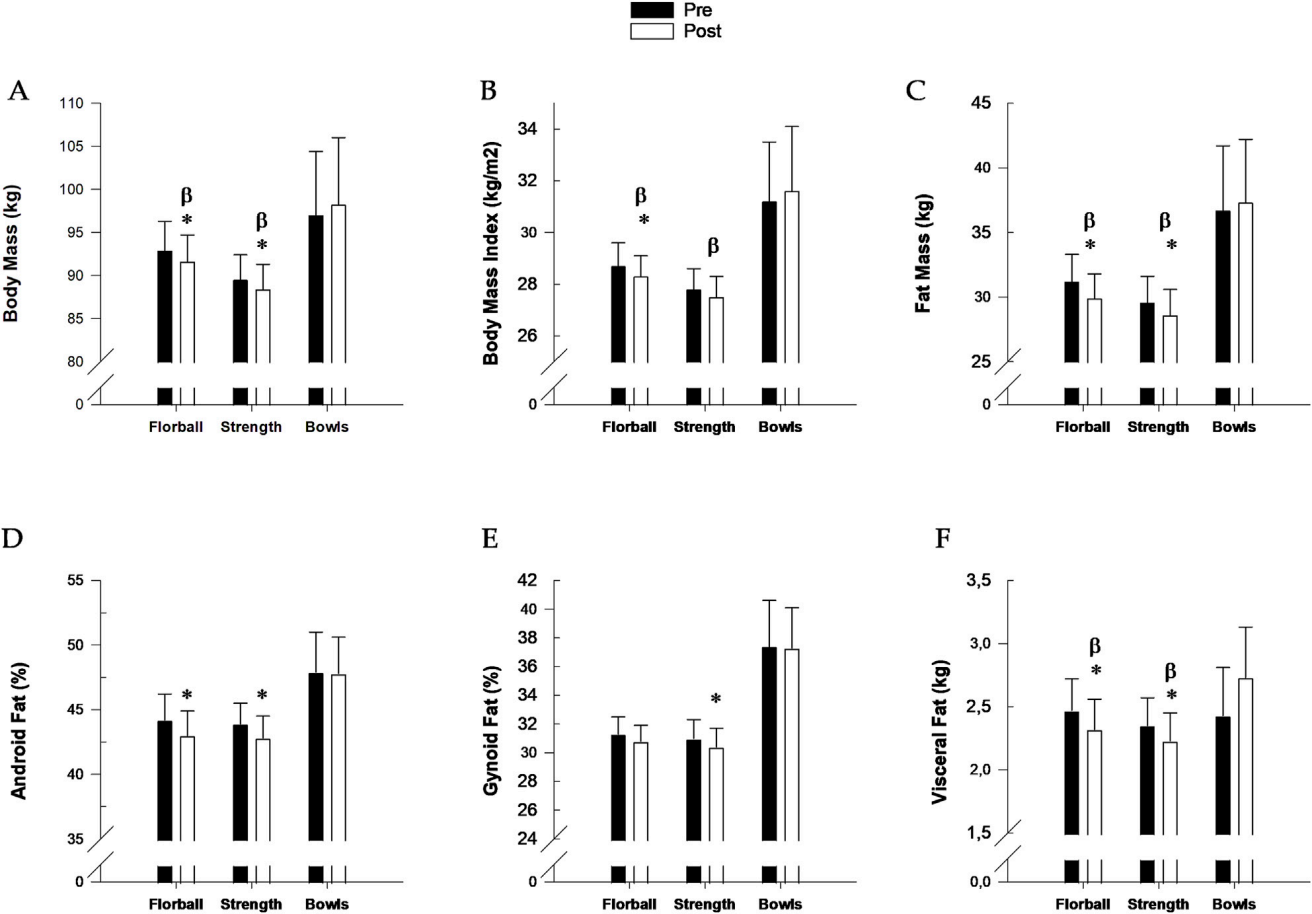
**(A)** Body mass; **(B)** Body mass index; **(C)** Fat mass; **(D)** Android fat mass; **(E)** Gynoid fat mass and **(F)** Visceral fat mass before (pre, filled bars) and after (post, open bars) 12 weeks of training floorball (n = 22), strength (n = 24) and bowls (n = 9). Data are presented as mean ± SE. * Significant (p < 0.05) within group change from baseline. ^β^Change significant (p < 0.05) different from change in Bowls.

Body mass decreased in FG (−1.4% ± 0.7%, P = 0.031) and SG (−1.3% ± 0.6%, P = 0.03) during INT, and the changes were different (P = 0.013 and P = 0.015, respectively) from BG, where no change was observed (Figure 3). BMI decreased in FG (−1.4% ± 0.7%, P = 0.03) and SG (−1.1 %± 0.7%, P = 0.05) with no change in BG. The change in BMI in FG and SG was different (P = 0.023 and P = 0.038) from that in BG. Fat mass and visceral fat decreased in FG (−3.9% ± 1.9%, P = 0.0.023; −6.3% ± 3.5%, P = 0.044) and SG (−3.4 %± 1.1%, P = 0.004; −5.2% ± 2.1%, P = 0.024) during INT, which were different (P = 0.04) from BG, where no changes were observed. Android fat decreased in FG (−6.9% ± 1.0%, P = 0.007) and SG (−3.4% ± 1.3%, P = 0.013) during INT, which were not different from BG, where no change was observed. Gynoid fat did not change in FG, but decreased in SG (−2.1% ± 0.8%, P = 0.007), which was not different from BG, where no change was observed (Figure 3). No change in total, leg and arm lean mass during INT was observed in any of the groups (Table 2). There were no differences between changes in FG and SG during INT for any of the measurements of body composition (Figure 3; Table 2).

**TABLE 2:** Blood glycosylated haemoglobin, glucose, High Density Lipoprotein, Low Density Lipoprotein and total cholesterol (A); total lean body mass, leg lean mass and arm lean mass (B) before (Pre) and after (Post) 12 weeks of floorball, strength and bowls training.

	Floorball (n = 22)		Strength (n = 24)		Bowls (n = 9)	
	Pre	Post	Pre	Post	Pre	Post
A						
Hb1Ac (mmol/mol)	36.4 ± 1.2	35.5 ± 1.0*	38.0 ± 0.9	35.3 ± 1.6*	38.3 ± 1.7	38.4 ± 1.3
Glucose (mmol/L)	6.0 ± 0.2	6.0 ± 0.2^β^	6.0 ± 0.2	6.0 ± 0.2^β^	7.2 ± 0.2	7.7 ± 0.4*
HDL cholesterol (mmol/L)	1.4 ± 0.1	1.5 ± 0.1	1.4 ± 0.1	1.6 ± 0.1*	1.3 ± 0.1	1.4 ± 0.1
LDL cholesterol (mmol/L)	3.5 ± 0.2	3.4 ± 0.1	3.9 ± 0.2	3.5 ± 0.2*	2.3 ± 0.4	2.2 ± 0.3
Total cholesterol (mmol/L)	5.2 ± 0.2	5.1 ± 0.2	5.6 ± 0.2	5.3 ± 0.2*	4.2 ± 0.4	4.3 ± 0.3
B						
Lean body mass (kg)	58.2 ± 1.5	58.2 ± 1.5	56.2 ± 1.2	56.3 ± 1.3	57.3 ± 3.0	58.2 ± 3.4
Leg lean mass (kg)	20.1 ± 0.6	20.1 ± 0.6	19.1 ± 0.5	19.2 ± 0.6	19.9 ± 1.1	20.2 ± 1.3
Arm lean mass (kg)	7.0 ± 0.2	7.0 ± 0.2	6.7 ± 0.2	8.0 ± 0.2	6.9 ± 0.5	6.9 ± 0.6

Values are mean ± SE. Hb1Ac, Glycosylated hemoglobin; HDL, high density lipoprotein; LDL, Low Density Lipoprotein. *Significant (p < 0.05) within group change from baseline. ^β^Change significant (p < 0.05) different from change in bowls training group.

#### 3.1.3 Plasma bone markers, bone mass and bone mass density

There was an increase in plasma P1NP (11.8% ± 5.2%, P = 0.017) in FG during INT, which was not different from SG and BG, where no changes were observed (Table 3). Plasma osteocalcin increased in FG (10.6% ± 4.8%, P = 0.017) during INT, which was different (P < 0.05) from a decrease in SG (−5.5% ± 5.2%, P = 0.049). The decrease in SG was different (P = 0.018) from BG, where no change was observed. For plasma CTX, there were no changes in FG, SG or BG during INT, Total, leg and arm bone mass, as well as total, leg and arm BMD did not change during INT in either group (Table 3).

**TABLE 3:** Blood bone markers (CTX, P1NP and osteocalcin), bone mass and Bone Mass Density before (Pre) and after (Post) 12 weeks of floorball, strength and bowls training. For bone markers analyses failed for three subjects in the floorball group and two subjects in the Bowls group leaving results only for 19 subjects in FG and seven subjects in BG for this parameter.

	Floorball (n = 22)		Strength (n = 24)		Bowls (n = 9)	
	Pre	Post	Pre	Post	Pre	Post
CTX (ng/L)	237.9 ± 35.4	249 ± 43	359 ± 44	332 ± 38	303 ± 76	330 ± 88
P1NP (µg/L)	41.8 ± 2.6	46.7 ± 4.0*	50.2 ± 3.3	50.1 ± 3.6	47.4 ± 8.8	49.5 ± 9.4
Osteocalcin (µg/L)	13.5 ± 0.8	15.0 ± 1.0*α	18.8 ± 1.8	17.8 ± 1.6*^,β,γ^	17.8 ± 3.4	19.8 ± 4.3α
Total bone mass (kg)	3.26 ± 0.10	3.26 ± 0.10	3.15 ± 0.06	3.13 ± 0.07	3.11 ± 0.14	3.09 ± 0.13
Leg bone mass (kg)	1.25 ± 0.04	1.24 ± 0.03	1.23 ± 0.03	1.22 ± 0.03	1.17 ± 0.04	1.18 ± 0.04
Arm bone mass (g)	0.49 ± 0.07	0.49 ± 0.08	0.47 ± 0.06	0.47 ± 0.07	397 ± 51	444 ± 23
Total BMD (g cm^2^)	1.33 ± 0.03	1.33 ± 0.02	1.32 ± 0.03	1.32 ± 0.02	1.29 ± 0.04	1.29 ± 0.04
Leg BMD (g cm^2^)	1.42 ± 0.03	1.41 ± 0.03	1.42 ± 0.02	1.41 ± 0.03	1.33 ± 0.14	1.33 ± 0.05
Arm BMD (g cm^2^)	1.10 ± 0.11	1.08 ± 0.10	1.06 ± 0.03	1.08 ± 0.02	1.05 ± 0.03	1.05 ± 0.03

Values are mean ± SE. CTX, Carboxy-terminal collagen crosslinks; P1NP, Serum/plasma procollagen-type 1 N propeptide; BMD, Bone Mass Density. *Significant (p < 0.05) within group change from baseline (p < 0.05). ^α^Change significant (p < 0.05) different from change in strength training group. ^β^Change significant (p < 0.05) different from change in bowls training group. ^γ^Change significant (p < 0.05) different from change in floorball training group.

#### 3.1.4 Plasma cholesterol

Plasma HDL cholesterol did not change in FG but increased in SG (13.2% ± 5.5%, P = 0.026) during INT, with the change not being different from BG, where no change was observed. For plasma LDL cholesterol, there was no change in FG during INT, but there was a decrease in SG (−9.9% ± 2.7%, P = 0.001), which was not different from no change in BG. Total plasma cholesterol did not change in FG during INT with SG having a decrease (−4.5 %± 2.0%, P = 0.04), which was not different from BG, where no change was observed (Table 2). There were no differences between changes in FG and SG during INT for any of the measurements of plasma cholesterol.

#### 3.1.5 Blood glycosylated hemoglobin and glucose

Hb1Ac decreased in FG (−2.6% ± 1.3%, P = 0.026) and SG (−3.5% ± 1.5%, P = 0.041) during INT (Table 2). The changes in FG and SG were not different from BG, where no change was observed. In FG and SG, no change in blood glucose was observed during INT, which were different (P = 0.012 and P = 0.006) from an increase in BG (7.7% ± 3.1%, P = 0.028) (Table 2).

#### 3.1.6 Heart rate and blood pressure at rest

**FIGURE 4:**
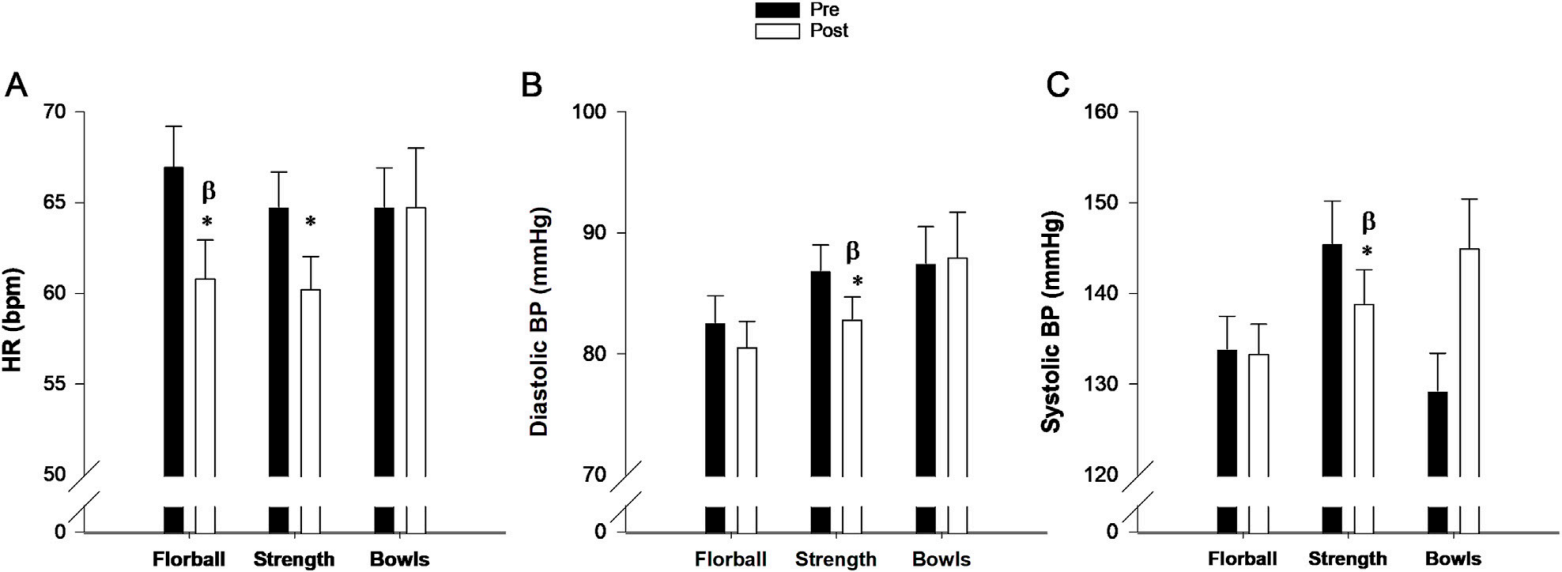
**(A)** Heart rate, **(B)** Diastolic blood pressure (BP) and **(C)** Systolic blood pressure (BP) at rest before (pre, filled bars) and after 3 months (post, open bars) of training floorball (n = 22), strength (n = 24) and bowls (n = 9). Data are presented as mean ± SE. * Significant (p < 0.05) within group change from baseline. ^β^Change significant (p < 0.05) different from changes in Bowls.

HR at rest decreased in FG (−9.2% ± 3.1%, P = 0.008) and SG (−6.9% ± 2.2%, P = 0.005) during INT, and the decrease in FG was different from BG, where no change was observed. Diastolic and systolic blood pressure did not change in FG but decreased in SG **(**-4.1% ± 1.9%, P = 0.004; −4.1% ± 1.9%, P = 0.03) during INT, which were different (P = 0.036 and P = 0.016) from BG, where no changes were observed (Figure 4).

There were no differences between changes in FG and SG for heart rate at rest and blood pressure (Figure 4).

#### 3.1.7 Functional measurements

**FIGURE 5:**
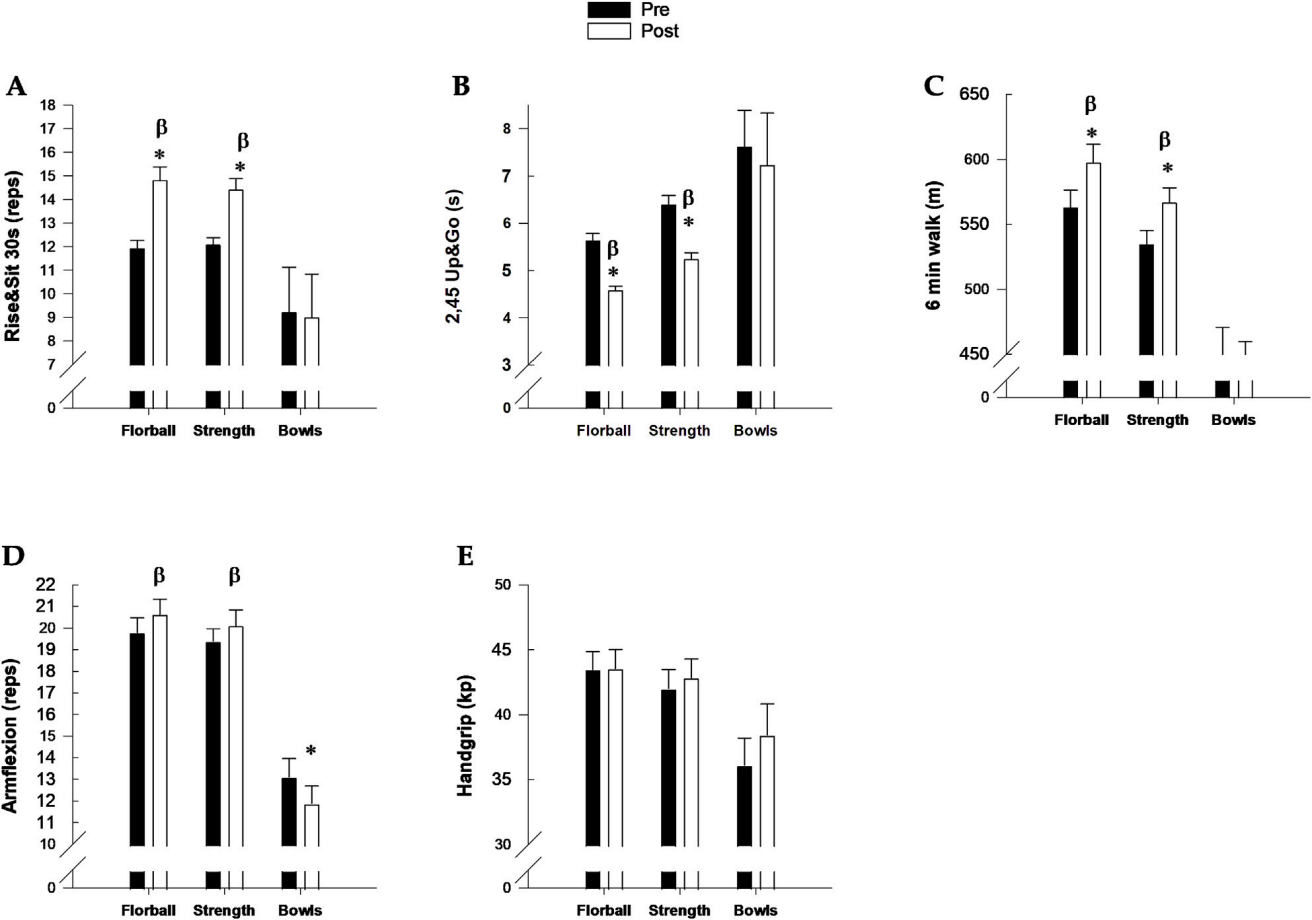
Functional tests. **(A)** Rise&Sit 30 s; **(B)** 2.45 Up&Go; **(C)** 6-min walk; **(D)** Arm flexion; **(E)** Handgrip before (pre, filled bars) and after (post, open bars) 12 weeks of training. Floorball (n = 29), Strength (n = 38) and Bowls (n = 9). Data are presented as mean ± SE. * Significant (p < 0.05) within group change from baseline. ^β^Change significant (p < 0.05) different from changes in Bowls.

The distance covered in 6 min and number of sit-to-stand repetitions in 30 s increased, and time for Timed Up and Go decreased in FG (6.1% ± 1.8%, P = 0.02; 24.3% ± 3.8%, P < 0.001; −18.9 %± 2.0%, P < 0.001) and SG (6.0% ± 1.7%, P = 0.001; 18.7 %± 2.5%, P < 0.00; **−**18.1 %± 2.8%, P < 0.001) during INT, which were different (P < 0.05) from BG, where no change was observed (Figure 5). The number of arm curls did not change in FG and SG during INT, whereas there was a decrease in BG (−9.3% ± 3.8%, P = 0.038) which was different from FG and SG (P = 0.006 and P = 0.009). Handgrip did not change in FG, SG or BG during INT. For none of the functional measurements differences between changes in FG and SG during INT were observed (Figure 5).

### 3.2 Effects of 24 weeks of training

For both FG and SG the changes after 12 weeks were maintained during the following 12-week training period (see the Supplementary Material).

## 4 Discussion

The major findings of the present study were that 12 weeks of floorball and strength training twice a week in elderly men lead to reduced blood Hb1Ac, body mass, fat mass, visceral and android fat with similar changes in the two groups. The strength training group also had a decrease in gynoid fat as well as in total and LDL plasma cholesterol. Furthermore, both groups had a decrease in heart rate at rest, and in the strength training group systolic and diastolic blood pressure were also reduced. As hypothesized, the floorball group had increase in markers for bone growth. Both groups had improvements in a number of the functional measurements with, as expected, similar responses. For all variables, the changes after 12 weeks were maintained during a following 12-week training period. The bowls group had no changes in any of the measured variables related to health and physical function.

### 4.1 Body composition

The older men in the present study had a BMI of ∼28 kg/m^2^, which classifies them as slightly obese. The men in both the floorball and strength training group lowered body mass by 1% during the 12-week intervention period, and reduced fat mass by 4% and 3%, respectively, which included decrease in visceral fat of 6% and 5% and android fat of 7% and 3%, respectively, as well as a 2%-lowering of gynoid fat in the strength training group. Likewise, Vorup et al. (2017b) found a reduction in total and visceral fat by 5% and 14%, respectively, but no reduction in body mass in older men (age: 70 years, BMI: 27 27 kg/m^2^) playing floorball 2 times 1 h a week for 12 weeks. Andersen et al. (2014), though, found a reduction in body mass of 1% following 12 weeks of football training twice a week for 60 min in elderly men with type 2 diabetes of a similar age and BMI (age: 68 years, BMI: 29 kg/m^2^) as the men in the present study, and their reduction in total fat mass and android fat mass was 4% and 8%, respectively.

The changes in body mass and fat mass obtained during the first 12 weeks of the training intervention were maintained during the following 12 weeks suggesting that the effects are sustained. In accordance (Pedersen et al., 2022), observed that 5 years of floorball training, reduced fat percentage, android and visceral fat by 10%, 13% and 18%, respectively, although body mass vas not changed. Furthermore, in the study by Andersen et al. (2016) with 52 weeks of football training body mass was significantly reduced (3%), whereas android and gynoid fat were non-significantly reduced (6.5% and 2.2%, respectively). Thus, team sport activities conducted as small-sided games appear effective in reducing fat mass.

High fat-percentage is associated with an increased risk of developing type 2 diabetes and cardiovascular diseases (Allison et al., 1997; Heitmann et al., 2000; Kyle et al., 2001; Anjana et al., 2004; Fox et al., 2007; Kang et al., 2011). In addition, visceral fat has been shown to be strongly associated with metabolic risk factors (Fox et al., 2007). Thus, the present results suggest that conducting regular floorball or strength training have positive effects on the health profile in older men.

Neither the floorball nor the strength training group had changes in lean body mass. This is in contrast to a study showing that muscle mass increased with two weekly 1-h sessions of small-sided soccer games for 12 weeks in young untrained adults (Krustrup et al., 2009). Vorup et al. (2017a) also found an increase in leg muscle mass (∼11%) after playing small sided team sport games (including 50% floorball) two times 20 min a week for 12 weeks in elderly men and women (age: 72 years; BMI: 26 kg/m^2^). However, in that study each training session was followed by ingestion of a drink with high protein content. Thus, it may be that a proper caloric and protein intake is necessary to increase muscle mass after team sport among elderly, as there was no change in a control group ingesting an isocaloric drink.

### 4.2 Blood variables

The subjects in the floorball group had no change in plasma total, LDL and HDL cholesterol, which is similar to findings in Vorup et al. (2017b) studying older men (age: 70 years, BMI: 27 kg/m^2^) playing floorball 2 times 1 h a week for 12 weeks. On the other hand, the subject in the strength training group had a reduction of plasma total cholesterol (5%) and LDL cholesterol (10%) as well as an increase in HDL cholesterol (13%), which were more pronounced compared to another study with strength training of elderly men (age: 66 years, BMI: 26 kg/m^2^) training once a week for 12 weeks (James et al., 2016; Chen et al., 2017), where the changes were −5%, −6% and 2%, respectively. Nevertheless, reduction in total and LDL cholesterol as well as increase in HDL cholesterol decrease the risk of coronary artery and peripheral vascular disease (Durstine et al., 2002). Thus, there were additional important adaptations for health promotion in the strength training group.

The subjects in the floorball group reduced blood Hb1Ac by 3%. Similarly, a study examining 12 weeks of 2 times 1 h of small-sided soccer training in men significant younger (50 years) than the men in the present study, but with diabetes mellitus Andersen et al. (2014), showed a reduction in blood HbA1c of 8%. On the other hand, Vorup et al. (2017b) did not find any significant change Hb1Ac in men (age: 70 years; BMI: 27 kg/m^2^) playing floorball 2 times 1 h a week for 12 weeks, but observed a reduction (18%) in insulin resistance determined by homeostatic model assessment (HOMA-IR). Thus, it appears that team sport conducted as small-sided games can have a positive effect on blood glucose control. It may be due to the high intensity actions and high heart rate during the training (Figure 2), as it was observed that men (age: 58 years; BMI 28 kg/m^2^) with type 2 diabetes (Andersen et al., 2014) conducting 10 weeks of high intensity interval 10-20-30 cycle training 3 times a week reduced HbA1c by 8%, whereas a similar group performing 150 min of moderate intensity exercise had no change in HbA1c(Baasch-Skytte et al., 2021).

In addition, the subjects in the strength training group lowered (4%) blood Hb1Ac. In accordance, Chen et al. (2017) showed that strength training 1 h a week for 12 weeks in elderly men (age: 66 years; BMI: 26 kg/m^2^) reduced blood HbA1c by 6% and blood glucose by 5%, and (James et al., 2016) found a reduction of 4% in blood glucose with 6 months of strength training 3 times 1 h a week among older men (age: 64 years; BMI: 26 kg/m^2^).

The lowering of blood HbA1c in both the floorball and strength training group is of clinical relevance, as a reduction by 1 percentage point lowers the risk of diabetes-related death by 21% (95% CI: 15%–27%) (Stratton et al., 2000).

### 4.3 Bone health

There was no change in BMD during the 12-week training intervention period in neither the floorball nor the strength training group. However, plasma markers for bone growth were higher (P1NP: 12%, osteocalcin: 5%) in the floorball group after 12 weeks of training indicating an increased rate of bone formation and turnover. This is in line with the finding by Helge et al. (2014), where 4 months of small-sided football training of elderly men (age: 68 years; BMI: 26 kg/m^2^) lead to an increase in plasma P1NP of 41% and osteocalcin of 45%. In that study leg BMD was not changed after 4 months but elevated by 5% after 12 months. Similarly, Pedersen et al. (2018) observed no changes in BMD after 12 weeks of floorball training twice a week among elderly men (age: 70 years; BMI: 27 kg/m^2^), but a significant increase in BMD after 2 years of floorball training which were maintained after 5 years with floorball training (Pedersen et al., 2022). In accordance, studies examining recreational football training of older men found higher leg BMD after 1 year of training (Kohrt et al., 2004; Helge et al., 2014). Apparently, team sport training does lead to increases in BMD when sustained.

No changes in plasma bone markers were observed in the strength or bowls training groups, except for a minimal reduction in osteocalcin in the strength training group, which could indicate a reduction in bone growth. As floorball and football are weight-bearing sports characterized by repeated intense bouts of sprints, accelerations, and decelerations bone growth is likely stimulated (Kohrt et al., 2004; Pedersen et al., 2018). In strength and bowls training no such activities are conducted, which may explain the lack of changes in plasma markers of bone turnover. Bolam et al. (2013) showed a gradual decline in BMD with age by an average of ∼0.7% per year after the age of 50 years. Thus, emphasizing the importance of conducting exercises that stimulate the bones. In that sense floorball and football training can be used, whereas strength training is less relevant.

### 4.4 Cardiovascular effects

Both the floorball and strength training group had lower heart rate at rest (9% and 7%, respectively) after compared to before the training intervention period, which indicates significant adaptations in the cardiovascular system (Reimers et al., 2018). This has also been observed in Vorup et al. (2017b) where a similar group of men (age: 70 years; BMI: 27 kg/m^2^) showed a reduction of 8% in heart rate at rest after a period with floorball training twice a week for 12 weeks. Likewise, a group of elderly men (age: 68 years; BMI: 26 kg/m^2^) conducting football training 2 to 3 times a week for 4 and 12 months lowered heart rate by 10% and 12%, respectively, which was associated with improvement in maximum oxygen uptake (VO2max) of 16% and 18%, respectively (Schmidt et al., 2014).

The floorball group had no change in blood pressure, which is in line with findings in the study by Vorup et al. (2017b) where elderly men were playing floorball twice a week for 12 weeks. On the other hand, the strength training group lowered systolic and diastolic blood pressure both by 4%, which is another sign of improved cardiovascular profile, and reduced risk of cardiovascular disease. In contrast, Schmidt et al. (2014) showed only minor cardiovascular adaptions in older men (age 68 years; BMI: 26 kg/m^2^) after both 4 and 12 months of strength training 2 to 3 times 1 h a week. Nevertheless, both floorball and strength training in older men appear to have positive cardiovascular effects.

### 4.5 Functional capacity

The distance covered in the 6-min walk test and number of sit-to-stand repetitions in 30 s (Rise&Sit 30 s) increased, and time for 2.45 Up&Go decreased in the floorball group (6%, 24% and 19%, respectively) during the 12-week intervention period (Figure 5). This is in line with findings in Duncan et al. (2022) who showed that 12 weeks of recreational small-sided football training twice a week among elderly men (age: 66 years; BMI: 29 kg/m^2^) resulted in improvement in the same functional tests (12%, 11% and 24%). Also, a study with elderly men (age: 69 years; BMI: 27 kg/m^2^) playing floorball two times 60 min a week for 12 weeks (Vorup et al., 2017b) showed improvements in the 6-min walk test (4%), but with no changes in the other two tests. After 5 years of floorball training, though, there were significant improvements in all three tests (4%, 8%, 4%) (Pedersen et al., 2022).

Also the strength training group had improvements in the 6-min walk, Rise&Sit 30 s and 2.45 Up&Go (6%, 19%, 18%) test during the intervention period (Figure 5). Similarly, Chen et al. (2017) studied the effect of 12 weeks of either eccentric or concentric strength training for 12 weeks of one session a week in elderly men (age: 66 years; BMI: 26 kg/m^2^) and found that both types of training lead to improvements in the 6-min walking (5% and 4%, respectively), Rise&Sit 30 s (37% and 18%) and 2.45 Up&Go (28% and 22%) test. Thus, both floorball and strength training do lead to increased functional capacity, which was maintained when continuing the training for another 12 weeks.

### 4.6 Limitations and strength

One of the limitations in the present study is that the subjects were not randomized to the various groups, which was chosen in order to optimize the motivation of the participants. It may have been one of the reasons that all subjects completed the training period with a compliance higher than 75%. Nevertheless, for most variables there were no differences between the groups before the intervention period, so the results were comparable. Furthermore, the used methods are valid and the results are clear, showing changes in, and some differences between, the floorball and strength training group, and for both groups significant differences compared to the bowls group, which functioned as a control group.

## 5 Conclusion

Older men conducting floorball and strength training twice a week for 12 weeks in a real-time setting improve functional capacity and a high number of health factors such as reducing heart rate at rest, body fat (in various compartments) and blood Hb1Ac. In addition, floorball training leads to bone growth and strength training lowers blood total and LDL cholesterol as well blood pressure. The effects can be maintained when continuing the training for another 12 weeks. In contrast, playing bowls does not lead to physiological changes.

## 6 Perspectives

The many positive effects on the health profile and improved functional capacity of taking part in floorball and strength training conducted in a setting where the older men were recruited through information in local newspapers look promising to use these activities to activate sedentary elderly men. Around half of the participant continued with the training after the first 12 weeks, and in questionaries the participants in the floorball group expressed as motivation for continuing that they feel engrossed in the activity highlighting the playful element and joy of playing together. In the strength training group, the main reason for continuing was that the strength training could be conducted individually and was flexible. Nevertheless, both training forms apparently are attractive for older men when they have a chance to get exposed to the activity.

## Data Availability

The original contributions presented in the study are included in the article/Supplementary Material, further inquiries can be directed to the corresponding author.
