# Supplementary material for: Effects of floorball and strength training in a real-life setting on health and physical function in older men
Source: Front Aging. 2025 Jul 25;6:1574084. doi: 10.3389/fragi.2025.1574084 (PMC12331617; doi:10.3389/fragi.2025.1574084)
Supplement: Supplementary file 1 [file Table1.docx]

**Supplementary Material**

**Table S1.** Variables at baseline and after 12 and 24 weeks of floorball and strength training for the floorball group and strength group.

|  | **Floorball (n=9)** | | |  | **Strength (n=13)** | | |
| --- | --- | --- | --- | --- | --- | --- | --- |
|  | Baseline | 12 weeks | 24 weeks |  | Baseline | 12 weeks | 24 weeks |
| Resting heart rate (bpm) | 67.3±2.1 | 65.6±3.9 | 61.2±2.2 |  | 67.4±2.1 | 60.7±3.2* | 66.3±3.2 |
| Diastolic BP (mmHG) | 82.4±2.9 | 81.0±2.2 | 83.3±2.8 |  | 86.7±2.1 | 83.0±4.6 | 86.4±2.2 |
| Systolic BP (mmHG) | 137±4.5 | 137±2.9 | 139±5.0 |  | 143±4.0 | 136±1.1 | 142±3.9 |
| Body Mass (kg) | 96.5±5.0 | 94.3±3.9 | 92.8±3.9* |  | 88.7±4.4 | 88.3±2.5 | 88.1±4.7 |
| Body mass Index (kg/m^2^) | 29.5±1.3 | 28.8±1.0 | 28.4±1.1 |  | 27.6±1.0 | 27.42.0 | 27.4±1.1 |
| Fat Mass (kg) | 33.0±2.9 | 30.9±1.8 | 29.7±1.4* |  | 28.3±2.7 | 27.3±*1.2 | 27.1±2.7* |
| Android Fat (%) | 46.5±1.9 | 44.5±1.5* | 43.3±1.4* |  | 42.2±2.0 | 41.2±*0.4 | 40.7±2.2* |
| Gynoid Fat (%) | 31.7±0.9 | 30.9±0.8 | 29.9±0.8* |  | 29.5±1.3 | 28.7±*1.2 | 28.2±1.1* |
| Visceral Fat (kg) | 2.68±0.36 | 2.36±0.24 | 2.24±0.25* |  | 2.35±0.35 | 2.27±0.35 | 2.27±0.38 |
| Tot Lean body mass (kg) | 59.6±2.3 | 59.6±2.3 | 59.6±2.4 |  | 57.3±1.7 | 57.8±2.0 | 57.9±2.0 |
| Leg lean body mass (kg) | 20.4±1.0 | 20.2±0.9 | 20.2±0.9 |  | 19.4±0.7 | 19.8 ± 0.8 | 19.5±0.8 |
| Arm lean body mass (kg) | 7.14±0.27 | 7.21±0.37 | 7.07±0.36 |  | 6.99±0.25 | 7.24±0.28 | 7.26±0.35 |
| Total bone mass (kg) | 3.46±0.15 | 3.47±0.15 | 3.44±0.15 |  | 3.09±0.08 | 3.07±0.09 | 3.09±0.09 |
| Leg bone mass (kg) | 1.31±0.05 | 1.31±0.05 | 1.30±0.05 |  | 1.21±0.03 | 1.2±0±0.03 | 1.21±0.03 |
| Arm bone mass (kg) | 0.46±0.06 | 0.52±0.93 | 0.51±0.02 |  | 0.43±0.04 | 0.48±0.02 | 0.48±0.02 |
| Total BMD (gcm^2^) | 1.40±0.04 | 1.38±0.04 | 1.38±0.04 |  | 1.27±0.03 | 1.27±0.03 | 1.26±0.03 |
| Leg BMD (g cm^2^) | 1.48±0.04 | 1.49±0.04 | 1.49±0.04 |  | 1.37±0.02 | 1.36±0.03 | 1.38±0.03 |
| Arm BMD (g cm^2^) | 1.16±.03 | 1.10±0.03 | 1.10±.04 |  | 1.05±.04 | 1.07±0.03 | 1.01±.03 |
| CTX (ng/l) | 216±41 | 220±56* | 242±49 |  | 376±59 | 327±57* | 341±57 |
| P1NP (µg/l) | 45.5±4.5 | 50.6±7.1* | 48.2±6.7 |  | 52.2±4.2 | 51.0±3.0* | 52.8±4.5 |
| Osteocalcin (µg/l) | 14.7±1.3 | 48.2±6.7 | 16.7±1.2 |  | 18.6±2.0 | 53.1±4.2 | 18.7±2.2 |
| HDL cholesterol (mmol/l) | 1.23±0.18 | 1.33±0.19 | 1.37±0.17 |  | 1.39±0.08 | 1.58±0.19 | 1.38±0.11 |
| LDL cholesterol (mmol/l) | 3.53±0.29 | 3.38±0.28 | 3.24±0.1 |  | 4.29±0.28 | 4.00±*0.21 | 3.68±0.26* |
| Total cholesterol (mmol/l) | 5.46±0.41 | 5.11± 0.32 | 5.05±0.26 |  | 5.94±0.26 | 5.72±0.22 | 5.44±0.29* |
| Hb1Ac (mmol/mol) | 5.62±0.21 | 5.48±0.18 | 5.66±0.23 |  | 5.71±0.12 | 5.71±0.22 | 5.78±0.13 |
| Glucose (mmol/l) | 6.01±0.39 | 6.04±0.29 | 5.98±0.32 |  | 6.19±0.25 | 6.08±0.20 | 6.32±0.36 |
| Maximal walk 6 min (m) | 553±18 | 591±22 | 591±25* |  | 565±12 | 590±15* | 613±17* |
| Rise&Sit 30s (reps) | 11.8±0.6 | 14.1±0.5* | 15.0±0.5* |  | 12.1±0.5 | 14.7±0.8* | 15.3±0.8* |
| 2.45 Up&Go (s) | 6.15±0.17 | 4.73±0.12 | 4.71±0.15* |  | 5.78±0.15 | 5.16±0.29* | 4.77±0.17* |
| Armflexion (reps) | 20.4±0.7 | 22.4±0.5* | 21.1±0.7 |  | 21.5±0.8 | 22.0±0.9 | 22.5±0.5 |
| Handgrip (kp) | 43.2±1.2 | 43.9±2.5 | 42.4±1.9 |  | 44.1±1.6 | 46.1±1.5 | 45.0±1.2 |

Values are mean ± SE. BP= Blood pressure, BMD= Bone mass density, CTX = Carboxy-terminal collagen crosslinks, P1NP = Serum/plasma procollagen-type 1 N propeptide, HDL = High Density Lipoprotein, LDL = Low Density Lipoprotein, Hb1Ac = glycosylated hemoglobin. *Significant (p<0.05) within group change from baseline.
